# A Rare Case of Cranial Nerve VII Neuropraxia Associated with Alveolar Nerve Blocks

**DOI:** 10.5811/cpcem.2021.4.51264

**Published:** 2021-05-10

**Authors:** Stephen Allegra, Richard Church, Veera Sudireddy

**Affiliations:** University of Massachusetts Memorial Medical Center, Department of Emergency Medicine, Worcester, Massachusetts

**Keywords:** Alveolar nerve block, Bell’s palsy, facial neuropraxia

## Abstract

**Case Presentation:**

A 26-year-old male presented to our emergency department for six days of right-sided facial myasthenia and parasthesias following a dental procedure using anesthetic nerve blocks.

**Discussion:**

Iatrogenic cranial nerve VII neuropraxia, a peripheral nerve injury, is an uncommon complication of alveolar nerve blocks with few documented cases specifically due to dental anesthesia. Treatment usually involves use of oral corticosteroid and/or antiviral medications along with close follow-up in clinic with a neurologist and/or otolaryngologist.

## CASE PRESENTATION

A 26-year-old male with no significant medical history presented to our emergency department for several days of persistent, right-sided facial numbness and weakness. He had visited the dentist six days prior for an extensive cleaning under local anesthesia, receiving right superior and inferior alveolar nerve blocks. He had immediate and unabated loss of motor function to the right side of his face after this procedure along with an inability to close his right eye and mouth. The patient denied any history of recent oral herpes lesions, rash, headaches, dysarthria, dysphagia, tick bites, outdoor activities, or recent eye, ear or oral infections. This patient’s history and physical exam findings suggested diagnosis.

## DISCUSSION

### Iatrogenic Right Cranial Nerve VII Neuropraxia

**Image 1:**
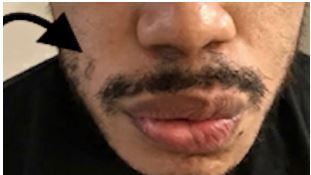
Flattening of the right nasolabial fold with inability to contract right-side of orbicularis oris muscle.

**Image 2:**
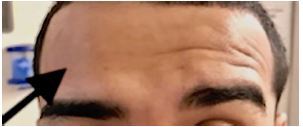
Inability to contract frontalis muscle on right side.

**Image 3:**
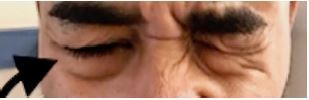
Inability to contract right orbicularis oculi muscle.

Cranial nerve (CN) VII palsy is a common physical exam finding; however, the presumed etiology of neuropraxia following a dental block is a rare complication, and images are not readily found in the emergency medicine literature.^1^ Bell’s palsy (or CN VII palsy) is described as an acute, unilateral peripheral nerve palsy that leads to temporary paralysis of the affected side of the face and forehead.^2^ On exam, the patient had right CN VII palsy involving the right forehead (Images 1–3). The neurology service was consulted, and recommendations were for initiation of oral prednisone and valacyclovir daily along with outpatient follow-up for magnetic resonance imaging of the brain and right facial nerve.

Although our patient had an acute onset of Bell’s palsy following a dental nerve block, the etiology is often uncertain and includes viral infection, autoimmune inflammatory disorders, inherited predisposition, and vascular ischemia.^1^–^4^ Patients should be given close follow-up with a neurologist and/or otolaryngologist for continued treatment and management as an outpatient with corticosteroid and antiviral medications.^3^,^5^

**CPC-EM Capsule**

What do we already know about this clinical entity?

*Iatrogenic seventh cranial nerve palsy is a rare procedural complication not well reported in the emergency medicine literature.*

What is the major impact of the image(s)?

*Neuropraxia following a dental block is a rare complication that requires close follow-up with a neurologist and/or otolaryngologist for continued management.*

How might this improve emergency medicine practice?

*Rapid identification will aid in treatment of an uncommon facial nerve palsy that can be managed in the outpatient setting.*
